# Non-contiguous finished genome sequence and description of *Anaerococcus vaginalis*

**DOI:** 10.4056/sigs.2716452

**Published:** 2012-07-23

**Authors:** Perrine Hugon, Ajay Kumar Mishra, Catherine Robert, Didier Raoult, Pierre-Edouard Fournier

**Affiliations:** 1 Unité de Recherche sur les Maladies Infectieuses et Tropicales Emergentes, UMR CNRS 6236 – IRD 198, Faculté de médecine, Aix-Marseille Université

**Keywords:** *Anaerococcus vaginalis*, genome

## Abstract

We report the properties of a draft genome sequence of the bacterium *Anaerococcus vaginalis* strain PH9, a species within the *Anaerococcus* genus. This strain, whose genome is described here, was isolated from the fecal flora of a 26-year-old woman suffering from morbid obesity. *A. vaginalis* is an obligate anaerobic coccus. Here we describe the features of this organism, together with the complete genome sequence and annotation. The 2,048,125-bp long (one chromosome but no plasmid) and contains 2,095 protein-coding and 38 RNA genes, including three rRNA genes.

## Introduction

*Anaerococcus vaginalis* strain PH9 (= CSUR P188= DSM25446) was isolated from the stool of a 26-year-old woman suffering from morbid obesity as part of a study aiming at cultivating all species within human feces. It is a Gram-positive, anaerobic, indole-negative coccus.

The genus *Anaerococcus* (Ezaki *et al*. 2001) was created in 2001 [1] and to date, this genus consist of saccharolytic, butyrate-producing anaerobic and non-motile gram-positive cocci. Seven species are validated, including *A. hydrogenalis*, *A. lactolyticus*, *A. murdochii, A. octavius*, *A. prevotii*, *A. tetradius* and *A. vaginalis* [2,3]. Members of the genus *Anaerococcus* have mostly been isolated from the human vagina, but have also occasionally been identified in the nasal cavity, on the skin, and in various infectious processes including ovarian, peritoneal, sacral, digital and cervical abscesses, vaginoses, bacteremias, foot ulcers, a sternal wound, and a knee arthritis [1-5]. In addition, uncultured bacteria with 16S rRNA sequences highly similar to members of the *Anaerococcus* genus have been detected in metagenomes from the human skin flora [6].

*A. vaginalis* (Li *et al*. 1992) was first isolated from vaginal discharges and ovarian abscesses [7]. Initially, it was classified in the genus *Peptostreptococcus* but later reclassified within the genus *Anaerococcus* [1].

To the best of our knowledge, we first report the isolation of *Anaerococcus sp.* from the fecal flora of a patient suffering from morbid obesity. Herein, we present a set of features for of *A. vaginalis* strain PH9 together with the description of the complete genomic sequence and annotation.

## Classification and features

A stool sample was collected from a 26-year-old woman living in Marseille, France, who suffered from morbid obesity: BMI=48.2 (118.8 kg, 1.57 meter). At the time of stool sample collection, she was not a drug-user and was not on a diet. The patient gave an informed and signed consent, and the agreement of local ethics committee of the IFR48 (Marseille, France) were obtained under agreement 11-017. The fecal specimen was preserved at -80°C after collection. Strain PH9 (Table 1) was isolated in 2011 by anaerobic cultivation on 5% sheep blood-enriched Columbia agar (BioMerieux, Marcy l’Etoile, France) after 4 days of preincubation of the stool sample with addition of thioglycolate in a blood culture bottle.

**Table 1:** Classification and general features of *Anaerococcus vaginalis* strain PH9

**MIGS ID**	**Property**	**Term**	**Evidence code^a^**
	Current classification	Domain *Bacteria*	TAS [8]
		Phylum *Firmicutes*	TAS [9-11]
		Class *Clostridia*	TAS [12,13]
		Order *Clostridiales*	TAS [14,15]
		Family *Clostridiales* family XI *Incertae sedis*	TAS [16]
		Genus *Anaerococcus*	TAS [1]
		Species *Anaerococcus vaginalis*	IDA
		Type strain PH9	
	Gram stain	positive	IDA
	Cell shape	coccoid	IDA
	Motility	nonmotile	IDA
	Sporulation	nonsporulating	IDA
	Temperature range	mesophile	IDA
	Optimum temperature	37°C	IDA
MIGS-6.3	Salinity	growth in BHI medium + 5% NaCl	IDA
MIGS-22	Oxygen requirement	anaerobic	IDA
	Carbon source	unknown	
	Energy source	peptones	NAS
MIGS-6	Habitat	human gut	IDA
MIGS-15	Biotic relationship	free living	IDA
MIGS-14	Pathogenicity Biosafety level Isolation	unknown 2 human feces	
MIGS-4	Geographic location	France	IDA
MIGS-5	Sample collection time	January 2011	IDA
MIGS-4.1	Latitude	43.296482	IDA
MIGS-4.1	Longitude	5.36978	IDA
MIGS-4.3	Depth	surface	IDA
MIGS-4.4	Altitude	0 m above sea level	IDA

Evidence codes – IDA: Inferred from Direct Assay; TAS: Traceable Author Statement (i.e., a direct report exists in the literature); NAS: Non-traceable Author Statement (i.e., not directly observed for the living, isolated sample, but based on a generally accepted property for the species, or anecdotal evidence). These evidence codes are from the Gene Ontology project [17]. If the evidence is IDA, then the property was directly observed for a live isolate by one of the authors or an expert mentioned in the acknowledgements.

**Figure 1:**
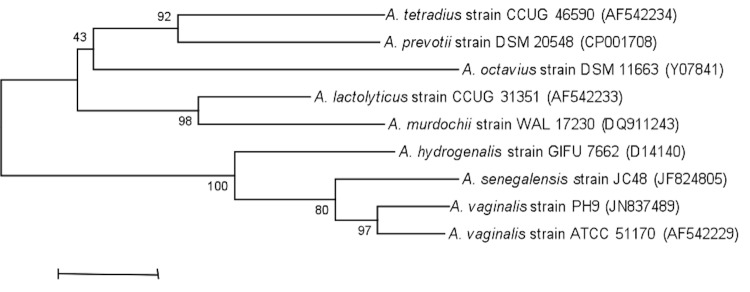
Phylogenetic tree highlighting the position of *Anaerococcus vaginalis* strain PH9 relative to other type strains within the *Anaerococcus* genus. GenBank accession numbers are indicated in parentheses. Sequences were aligned using CLUSTALW, and phylogenetic inferences obtained using the maximum-likelihood method and the MEGA software. Numbers at the nodes are bootstrap values obtained by repeating 500 times the analysis to generate a majority consensus tree. The scale bar represents a 1% nucleotide sequence divergence.

This strain exhibited a 98.8% 16S rRNA sequence similarity with *A. vaginalis* (Li *et al*. 1992), the phylogenetically closest validated *Anaerococcus* species (Figure 1). This value was higher than the 98.7% 16S rRNA gene sequence threshold recommended by Stackebrandt and Ebers to delineate a new species [18]. As a consequence, strain PH9 belongs to *A. vaginalis*.

**Figure 2:**
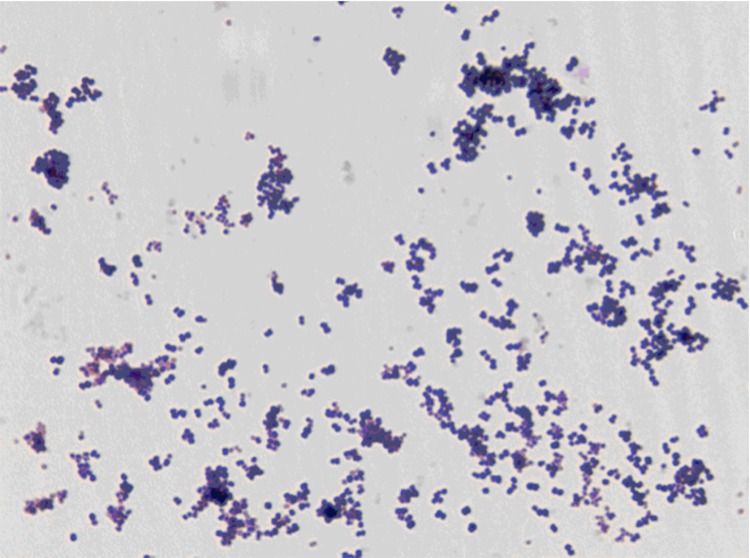
Gram staining of *A. vaginalis* strain PH9

**Figure 3:**
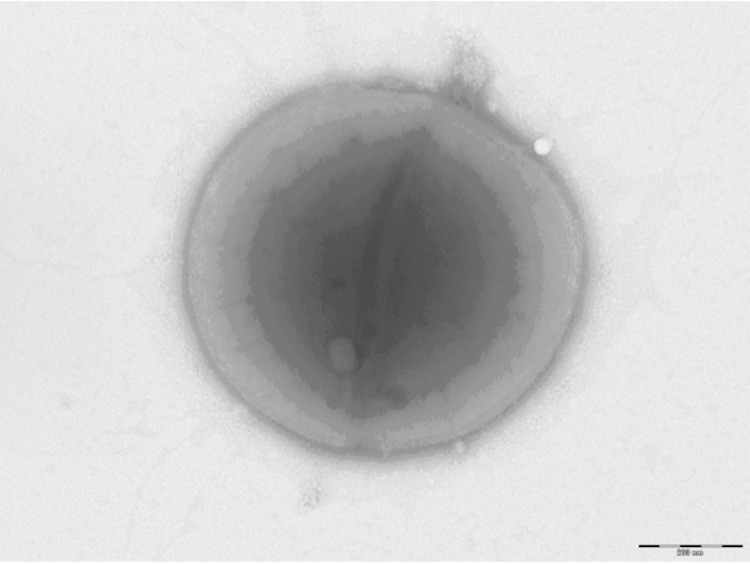
Transmission electron microscopy of *A. vaginalis* strain PH9, using a Morgani 268D (Philips) at an operating voltage of 60kV.The scale bar represents 900 nm.

Growth at different temperatures (25, 30, 37, 45°C) was tested; no growth occurred at 25°C and 45°C, growth occurred between 30 and 37°C, and optimal growth was observed at 37°C. Colonies were 0.5 mm to 1 mm in diameter on blood-enriched Columbia agar and Brain Heart Infusion (BHI) agar. Growth of the strain was tested under anaerobic and microaerophilic conditions using GENbag anaer and GENbag microaer systems, respectively (BioMérieux), and in the presence of air, with or without 5% CO_2_ and in aerobic conditions. Optimal growth was obtained anaerobically, with weak growth being observed in microaerophilic condition, and no growth occurring in aerobic conditions and with 5% CO_2_. Gram staining showed Gram positive cocci. A motility test was negative. Cells grown on agar are Gram-positive (Figure 2) and have a mean diameter of 0.71 µm by electron microscopy and are mostly grouped in pairs, short chains or small clumps (Figure 3).

Strain PH9 exhibited catalase activity but no oxidase activity. Using API Rapid ID 32A, a positive reaction was observed for arginine dihydrolase, histidine arylamidase, leucine arylamidase and mannose fermentation. A weak activity was observed for glycine arylamidase. *A. vaginalis* is susceptible to penicillin G, imipeneme, amoxicillin + clavulanic acid, vancomycin, clindamycin and metronidazole.

**Figure 4:**
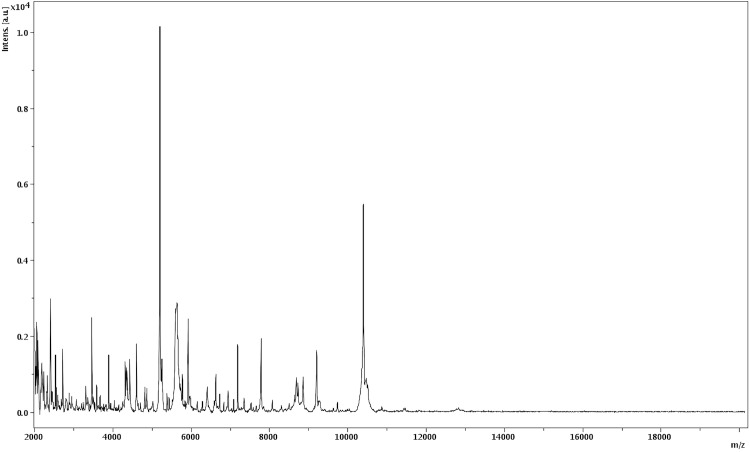
Reference mass spectrum from *A. vaginalis* strain PH9. Spectra from 12 individual colonies were compared and a reference spectrum was generated.

Matrix-assisted laser-desorption/ionization time-of-flight (MALDI-TOF) MS protein analysis was carried out as previously described [19]. Briefly, a pipette tip was used to pick one isolated bacterial colony from a culture agar plate, and to spread it as a thin film on a MTP 384 MALDI-TOF target plate (Bruker Daltonics, Germany). Twelve distinct deposits were done for strain PH9 from twelve isolated colonies. Each smear was overlaid with 2µL of matrix solution (saturated solution of alpha-cyano-4-hydroxycinnamic acid) in 50% acetonitrile, 2.5% tri-fluoracetic acid, and allowed to dry for five minutes. Measurements were performed with a Microflex spectrometer (Bruker). Spectra were recorded in the positive linear mode for the mass range of 2,000 to 20,000 Da (parameter settings: ion source 1 (ISI), 20kV; IS2, 18.5 kV; lens, 7 kV). A spectrum was obtained after 675 shots at a variable laser power. The time of acquisition was between 30 seconds and 1 minute per spot. The twelve PH9 spectra were imported into the MALDI Bio Typer software (version 2.0, Bruker) and analyzed by standard pattern matching (with default parameter settings) against the main spectra of 2,843 bacteria, including spectra from seven validated *Anaerococcus* species used as reference data, in the Bio Typer database. The method of identification includes the m/z from 3,000 to 15,000 Da. For every spectrum, 100 peaks at most were taken into account and compared with the spectra in the database. A score enabled the presumptive identification and discrimination of the tested species from those in the database: a score ≥ 2 with a validated species enabled the identification at the species level; a score ≥ 1.7 but < 2 enabled the identification at the genus level; and a score < 1.7 did not enable any identification. Spectra were compared with the Bruker database that contained spectra from the seven validated *Anaerococcus* species. The score and spectra obtained were similar to those of *A. vaginalis*, thus confirming that our isolate was a member of the *A. vaginalis* species. We incremented our database with the spectrum from strain PH9 (Figure 4).

## Genome sequencing and annotation

### Genome project history

The organism was selected for sequencing on the basis of its phylogenetic position and 16S rRNA similarity to other members of the *Anaerococcus* genus, and is part of a study of the human digestive flora aiming at isolating all bacterial species within human feces. It is the third published genome from an *Anaerococcus* species and the first genome from the *A. vaginalis* species. A summary of the project information is shown in Table 2. The Genbank accession number is CAGU00000000. The genome consists of 93 contigs. Table 2 shows the project information and its association with MIGS version 2.0 compliance [1].

**Table 2:** Project information

**MIGS ID**	**Property**	**Term**
MIGS-31	Finishing quality	High-quality draft
MIGS-28	Libraries used	One 454 paired end 3-kb library
MIGS-29	Sequencing platforms	454 GS FLX Titanium
MIGS-31.2	Fold coverage	35
MIGS-30	Assemblers	Newbler version 2.5.3
MIGS-32	Gene calling method	Prodigal
	INSDC ID	2000019206
	NCBI project ID	CAGU00000000
	Genbank Date of Release	31-05-2012
	Gold ID	Gi13719
MIGS-13	Project relevance	Study of the human gut microbiome

### Growth conditions and DNA isolation

*A. vaginalis* strain PH9 (DSM25446, CSUR P188) was grown anaerobically on 5% sheep blood-enriched Columbia agar at 37°C. Six petri dishes were spread and resuspended in 6x100µl of G2 buffer (EZ1 DNA Tissue kit, Qiagen). A first mechanical lysis was performed by glass powder on the Fastprep-24 device (Sample Preparation system from MP Biomedicals, USA) for 40 seconds. DNA was then incubated for a lysozyme treatment (30 minutes at 37°C) and extracted using the BioRobot EZ 1 Advanced XL (Qiagen).The DNA was then concentrated and purified using the Qiamp kit (Qiagen). The yield and the concentration was measured by the Quant-it Picogreen kit (Invitrogen) on the Genios_Tecan fluorometer at 115.2ng/µl.

### Genome sequencing and assembly

Five µg of DNA were mechanically fragmented on the Hydroshear device (Digilab, Holliston, MA,USA) with an enrichment size at 3-4kb. The DNA fragmentation was visualized through the Agilent 2100 BioAnalyzer on a DNA labchip 7500 with an optimal size of 2.92 kb. The library was constructed according to the 454 GS FLX Titanium paired end protocol. Circularization and nebulization were performed and generated a pattern with an optimal at 415 bp. After PCR amplification through 15 cycles followed by double size selection, the single stranded paired-end library was then quantified on the Quant-it Ribogreen kit (Invitrogen) on the Genios Tecan fluorometer at 1,440 pg/µL. The library concentration equivalence was calculated as 6.36E+09 molecules/µL. The library was stored at -20°C until further use.

The library was clonally amplified with 0.25cpb and 1cpb respectively in 2x8 emPCR reactions with the GS Titanium SV emPCR Kit (Lib-L) v2 (Roche). The yields of the emPCR were quite high at 17.78% but in the range of 5 to 20% from the Roche procedure.

Approximately 790,000 beads were loaded on the GS Titanium PicoTiterPlate PTP Kit 70x75 and sequenced with the GS FLX Titanium Sequencing Kit XLR70 (Roche). The run was performed overnight and then analyzed on the cluster through the gsRunBrowser and Newbler assembler (Roche). A total of 191,750 passed filter wells were obtained and generated 59.42 Mb with an average length of 309 bp. The passed filter sequences were assembled Using Newbler with 90% identity and 40bp as overlap. The final assembly identified 93 large contigs (>1500bp).

### Genome annotation

Open Reading Frames (ORFs) were predicted using Prodigal [20] with default parameters but the predicted ORFs were excluded if they were spanning a sequencing GAP region. The predicted bacterial protein sequences were searched against the GenBank database [21] and the Clusters of Orthologous Groups (COG) databases using BLASTP. The tRNAScanSE tool [22] was used to find tRNA genes, whereas ribosomal RNAs were found by using RNAmmer [23] and BLASTn against the NR database. ORFans were identified if their BLASTP *E*-value were lower than 1e-03 for alignment length greater than 80 amino acids. If alignment lengths were smaller than 80 amino acids, we used an *E*-value of 1e-05. Such parameter thresholds have already been used in previous works to define ORFans. To estimate the mean level of nucleotide sequence similarity at the genome level between *Anaerococcus* species, we compared the ORFs only using BLASTN and the following parameters: a query coverage of ≥ 70% and a minimum nucleotide length of 100 bp.

## Genome properties

**Figure 5:**
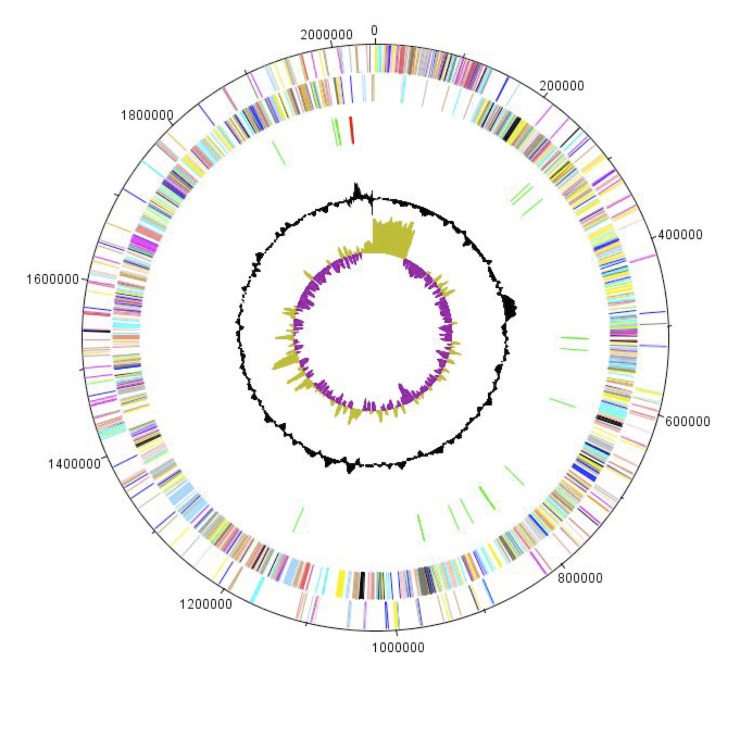
Graphical circular map of the chromosome. From outside to the center: Genes on the forward strand (colored by COG categories), genes on the reverse strand (colored by COG categories), RNA genes (tRNAs green, rRNAs red), GC content, and GC skew.

The genome of *A. vaginalis* strain PH9 is 2,048,125 bp long (1 chromosome, but no plasmid) with a 29.6% G + C content of (Figure 5 and Table 3). Of the 2,133 predicted genes, 2,095 were protein-coding genes, and 38 were RNAs. Three rRNA genes (one 16S rRNA, one 23S rRNA and one 5S rRNA) and 35 predicted tRNA genes were identified in the genome. A total of 1,546 genes (72.48%) were assigned a putative function. Eighty-one genes were identified as ORFans (3.8%). The remaining genes were annotated as hypothetical proteins. The properties and the statistics of the genome are summarized in Table 3. The distribution of genes into COGs functional categories is presented in Table 4.

**Table 3:** Nucleotide content and gene count levels of the genome

Attribute	Value	% of total^a^
Genome size (bp)	2,048,125	
DNA coding region (bp)	1,825,389	89.12
DNA G+C content (bp)	593,956	29.60
Total genes	2,133	100
RNA genes	38	1.78
Protein-coding genes	2,095	98.21
Genes with function prediction	1,556	72.94
Genes assigned to COGs	1,546	72.48
Genes with peptide signals	83	3.89
Genes with transmembrane helices	476	22.31

^a^The total is based on either the size of the genome in base pairs or the total number of protein coding genes in the annotated genome

**Table 4:** Number of genes associated with the 25 general COG functional categories

**Code**	**Value**	**%age**^a^	**Description**
J	144	6.87	Translation
A	0	0	RNA processing and modification
K	138	6.59	Transcription
L	146	6.97	Replication, recombination and repair
B	1	0.05	Chromatin structure and dynamics
D	19	0.91	Cell cycle control, mitosis and meiosis
Y	0	0	Nuclear structure
V	74	3.53	Defense mechanisms
T	47	2.24	Signal transduction mechanisms
M	66	3.15	Cell wall/membrane biogenesis
N	5	0.24	Cell motility
Z	0	0	Cytoskeleton
W	0	0	Extracellular structures
U	26	1.24	Intracellular trafficking and secretion
O	63	3.01	Posttranslational modification, protein turnover, chaperones
C	90	4.30	Energy production and conversion
G	123	5.87	Carbohydrate transport and metabolism
E	136	6.49	Amino acid transport and metabolism
F	60	2.86	Nucleotide transport and metabolism
H	59	2.82	Coenzyme transport and metabolism
I	33	1.58	Lipid transport and metabolism
P	96	4.58	Inorganic ion transport and metabolism
Q	24	1.15	Secondary metabolites biosynthesis, transport and catabolism
R	206	9.83	General function prediction only
S	123	5.87	Function unknown
-	542	25.41	Not in COGs

^a^The total is based on the total number of protein coding genes in the annotated genome.

## Comparison with the genomes from other *Anaerococcus* species

To date, two genomes from *Anaerococcus* species have been published. Here, we compared the genome sequence of *A. vaginalis* strain PH9 with those of *A. prevotii* strain PC1^T^ [24] and *A. senegalensis* strain JC48^T^ [25].

The draft genome sequence of *A. vaginalis* has a similar size to that of *A. prevotii* (2.04 *vs* 1.99 Mb, respectively), but a slightly larger than *A. senegalensis* (1.79Mb). The G+C content of *A. vaginalis* is comparable to *A. senegalensis* (29.60 *vs* 28.56%, respectively) and smaller than that of *A. prevotii* (35.64%). The gene content of *A. vaginalis* is larger than those of *A. prevotii* and *A. senegalensis* (2,133, 1,916 and 1,774, respectively). The ratio of genes per Mb of *A. vaginalis* is larger to those of *A. senegalensis* and *A. prevotii* (1,045, 991 and 962, respectively). Moreover, the distribution of genes into COG categories (Table 4) was highly similar in the three genomes.

*A. vaginalis* shared a mean 84.8% (range 71.10-100%) and 88.38% (range 70.3-100%) sequence similarity with *A. prevotii* and *A. senegalensis* respectively at the genome level.

## Conclusion

We describe the phenotypic, phylogenetic and genomic characteristics of *Anaerococcus vaginalis* strain PH9. This bacterial strain has been found in Marseille, France.

### Nucleotide sequence accession numbers

The *A. vaginalis* strain PH9 whole-genome shotgun (WGS) project and 16SrRNA gene sequence have been deposited in GenBank under accession numbers CAGU00000000 and JN837489, respectively.
